# Expression and Distribution of Neuropeptide-Expressing Cells Throughout the Rodent Paraventricular Nucleus of the Thalamus

**DOI:** 10.3389/fnbeh.2020.634163

**Published:** 2021-01-14

**Authors:** Genevieve R. Curtis, Kathleen Oakes, Jessica R. Barson

**Affiliations:** Department of Neurobiology and Anatomy, Drexel University College of Medicine, Philadelphia, PA, United States

**Keywords:** anterior, lateral, medial, motivated behavior, mouse, posterior, rat

## Abstract

The paraventricular nucleus of the thalamus (PVT) has been shown to make significant contributions to affective and motivated behavior, but a comprehensive description of the neurochemicals expressed in the cells of this brain region has never been presented. While the PVT is believed to be composed of projection neurons that primarily use as their neurotransmitter the excitatory amino acid, glutamate, several neuropeptides have also been described in this brain region. In this review article, we combine published literature with our observations from the Allen Brain Atlas to describe in detail the expression and distribution of neuropeptides in cells throughout the mouse and rat PVT, with a special focus on neuropeptides known to be involved in behavior. Several themes emerge from this investigation. First, while the majority of neuropeptides are expressed across the antero-posterior axis of the PVT, they generally exist in a gradient, in which expression is most dense but not exclusive in either the anterior or posterior PVT, although other neuropeptides display somewhat more equal expression in the anterior and posterior PVT but have reduced expression in the middle PVT. Second, we find overall that neuropeptides involved in arousal are more highly expressed in the anterior PVT, those involved in depression-like behavior are more highly expressed in the posterior PVT, and those involved in reward are more highly expressed in the medial PVT, while those involved in the intake of food and drugs of abuse are distributed throughout the PVT. Third, the pattern and content of neuropeptide expression in mice and rats appear not to be identical, and many neuropeptides found in the mouse PVT have not yet been demonstrated in the rat. Thus, while significantly more work is required to uncover the expression patterns and specific roles of individual neuropeptides in the PVT, the evidence thus far supports the existence of a diverse yet highly organized system of neuropeptides in this nucleus. Determined in part by their location within the PVT and their network of projections, the function of the neuropeptides in this system likely involves intricate coordination to influence both affective and motivated behavior.

## Introduction

The paraventricular nucleus of the thalamus (PVT) has been shown to make significant contributions to affective and motivated behavior (for review, see Kirouac, 2015; Millan et al., 2017; Barson et al., 2020; McGinty and Otis, 2020), but a comprehensive description of the neurochemicals expressed in the cells of this brain region has never been presented. The dorsal-most member of the midline thalamic nuclei, which receive dense afferent input from neuropeptide-containing neurons, the PVT itself is believed to be composed of projection neurons that primarily use as their neurotransmitter the excitatory amino acid, glutamate (Kirouac, 2015). Interestingly, however, several neuropeptides have also been described in this brain region. In this review article, we combine published literature with our observations from the Allen Brain Atlas (Allen Institute, 2004), to describe in detail the expression and distribution of neuropeptides in cells throughout the mouse and rat PVT.

Neuropeptides differ from the small molecule, classical neurotransmitters in their synthesis, release, and signaling. In neurons, neuropeptides always co-exist with one or more classical neurotransmitters and they can also be co-expressed with other neuropeptides (Hokfelt et al., 2018). Several neuropeptides have been identified not only in neurons but also in glial cells (Hokfelt et al., 2018). In contrast to neurotransmitters, neuropeptides are derived from larger, inactive molecule precursors that are synthesized in the endoplasmic reticulum and transferred to the Golgi apparatus for packaging into large, dense-core vesicles (Hokfelt et al., 2000). The bioactive peptide is then cleaved within the dense core vesicles by convertases, processing enzymes, and transported down the axon (Hokfelt et al., 2000). Release of neuropeptides into the extracellular space is triggered by small elevations in the calcium concentration in the bulk cytoplasm, rather than large elevations near the active zone at the synapse, and neuropeptides then bind to G protein-coupled receptors, which have affinities in the low nanomolar range, such that even a small number of peptide molecules can lead to effective signaling (Hokfelt et al., 2000, 2018). In contrast to neurotransmitters, neuropeptides are degraded by peptidases or diffuse away from the extracellular space, but they do not appear to have a reuptake mechanism (Hokfelt et al., 2000, 2018). Thus, while signaling can be very effective, replacement of neuropeptides can take considerable time. In cells of the PVT, it is very likely that most neuropeptides co-exist with the neurotransmitter, glutamate, and they may also be co-expressed with other neuropeptides.

The PVT extends through a relatively long antero-posterior axis (more than 2.1 mm in the adult mouse and 3.2 mm in the adult rat; Paxinos and Franklin, 2004; Paxinos and Watson, 2005) and, for this reason, researchers have examined subregions of this nucleus for differences in their neuroanatomical connections, behavioral function, and cellular composition. Most commonly, the PVT is divided into anterior and posterior subregions and, while these subregions project to many of the same brain regions, differences have been identified in the density of their efferent projections (Barson et al., 2020). For example, while the anterior PVT projects widely to other limbic areas, with denser projections to the suprachiasmatic nucleus of the hypothalamus, the posterior PVT has denser projections to the bed nucleus of the stria terminalis (BNST) and central nucleus of the amygdala (Moga and Moore, 1997; Li and Kirouac, 2008; Vertes and Hoover, 2008; Dong et al., 2017). Similarly, while most neurons across the PVT project to the nucleus accumbens, the anterior PVT has denser projections to the dorsomedial nucleus accumbens shell, while the posterior PVT has denser projections to the ventromedial shell (Dong et al., 2017). Rather than displaying a clear subregional division, however, these projections appear to have an antero-posterior gradient, as neurons projecting to the dorsomedial shell show a progressive decrease from the anterior to the posterior PVT, while those projecting to the ventromedial shell show a gradient in the opposite direction (Dong et al., 2017). Although it has been less well-documented, subregional distinctions in the PVT may also extend beyond anterior vs. posterior and also include medial vs. lateral and dorsal vs. ventral. For example, PVT neurons that project to the nucleus accumbens core are located in more lateral and ventral regions of the PVT than those that project to the shell (Dong et al., 2017). What these subregional distinctions could mean for behavior is still being explored. It has been suggested that the anterior PVT is more involved in behaviors with a positive emotional valence while the posterior PVT instead is more involved in those with negative valence (Dong et al., 2017). Alternatively, we have recently proposed that the anterior PVT is more involved in arousal while the posterior PVT is more involved in valence (Barson et al., 2020). Understanding the distribution of neuropeptides within the PVT, considered in light of their known roles in behavior, could offer more clues to its functional organization.

The purpose of this review article is to describe in detail the distribution of neuropeptides in cells throughout the rodent PVT, with a special focus on neuropeptides known to be involved in behavior. Based on our examination of this neuropeptide distribution, and acknowledging that there are exceptions, we propose that the anterior PVT is more involved in arousal while the posterior PVT is more involved in depression-like behavior, that the medial PVT is particularly involved in reward, and that the entire PVT affects the intake of food and drugs of abuse.

## Distribution and Function of Neuropeptides in the Paraventricular Thalamus

To identify neuropeptides in the rodent PVT, we used *in situ* hybridization data from the Allen Brain Atlas (Allen Institute, 2004). We used gray matter as a contrast and an expression threshold of 0.1, to analyze approximately 14,000 genes expressed in the PVT of the mouse brain. We identified 41 unique neuropeptides in this list, and used the Allen Brain Reference Atlas, Mouse Coronal v1 (Allen Institute, 2008); Allen Brain Reference Atlas, Mouse P56, Coronal (Allen Institute, 2011); and the Paxinos and Franklin mouse brain atlas (Paxinos and Franklin, 2004) to verify their location in the PVT. From this, we examined the gene expression of every neuropeptide in the coronal brain slices provided. The number of slices containing the PVT varied for each gene, ranging from 7 to 20, with an average of 11.5 slices per neuropeptide. All slices were from adult male C57Bl/6 mice. Of the 41 neuropeptides that we identified in the PVT, we classified 24 as being related to behavior (Table 1), 14 as related to cell function (Table 2), and three as related to reproduction (Table 3). For the neuropeptides that were specifically associated with affective and motivated behavior, we then searched the published literature to determine if and how these neuropeptides were previously described in the mouse and rat. In the following sections, we describe the distribution of nine of these neuropeptides, identified in the PVT and associated with affective and motivated behavior, and we also address their known or likely roles in this nucleus. For comparison, we describe the distribution of markers for glutamate and GABA in the PVT.

**Table 1:** Neuropeptides in the paraventricular nucleus of the thalamus (PVT) with known roles in behavior, as identified with the Allen Brain Atlas.

Behavior neuropeptides			
Gene	Peptide	Fold change vs. gray matter	Signal transduction pathway
*Tac2*	Neurokinin B	8.943	Gq/G11
*Gal*	Galanin peptides	5.273	Gq/G11, Gi/Go
*Agt*	Angiotensin	4.014	Gi/Go, Gq/G11
*Cck*	Cholecystokinin	1.555	Gq/G11, Gs
*Nts*	Neurotensin, neuromedin N	1.176	Gq/G11, Gs, Gi/Go
*Uts2b*	Urotensin-2B	1.077	Gq/G11
*Nucb2*	Nesfatin-l	1.058	Unknown
*Calca*	Calcitonin	1.003	Gs, Gq/G11, Gi/Go
*Adcyap1*	Pituitary adenylate cyclase-activating polypeptide	0.960	Gs, Gq/G11
*Calcb*	Calcitonin gene-related peptide	0.920	Gs, Gq/G11, Gi/Go
*Avp*	Vasopressin, neurophysin II, copeptin	0.860	Gq/G11, Gs
*Pdyn*	Dynorphins	0.852	Gi/Go, G12/G13
*Apln*	Apelins	0.786	Gi/Go
*Pomc*	Melanocyte-stimulating hormones, endorphins, adrenocorticotropic hormone, lipoproteins, corticotropin-like intermediate peptide	0.708	Gs, Gi/Go, Gq/G11
*Npvf*	Neuropeptide VF, neuropeptide SF	0.670	Gi/Go
*Grp*	Gastrin-releasing peptides, neuromedin C	0.648	Gq/G11
*Gcg*	Glucagon, glucagon-like peptides, and related peptides	0.637	Gs
*Pnoc*	Nociceptin, nocistatin	0.498	Gi/Go
*Cart*	Cocaine- and amphetamine-regulated transcript	0.453	Gi/Go
*Ucn*	Urocortin I	0.444	Gs, Gq/G11
*Crh*	Corticotropin-releasing hormone	0.353	Gs, Gq/G11
*Penk*	Enkephalins	0.203	Gs, Gq/G11
*Tac1*	Substance P, neurokinin A, neuropeptide K, neuropeptide gamma	0.167	Gs, Gq/G11
*Vip*	Vasoactive intestinal peptides	0.159	Gs, Gi/Go, Gq/G11

*Gene is the gene symbol; peptide is the known peptide(s) derived from the gene; fold change vs. gray matter is the degree of neuropeptide expression compared to overall gray matter in the brain; signal transduction pathway is the putative signaling activated by ligand binding to its receptor(s), as indicated by the International Union of Basic and Clinical Pharmacology (IUPHAR) and the British Pharmacological Society (BPS; Armstrong et al., 2019)*.

**Table 2:** Neuropeptides in the PVT with known roles in cell function, as identified with the Allen Brain Atlas.

Cell function neuropeptides		
Gene	Peptide	Fold change vs. gray matter
*Cbln4*	Cerebellin-4	5.693
*Nxphl*	Neurexophilin-l	3.534
*Cbln2*	Cerebellin-2	2.302
*Scg2*	Secretogranin II	1.888
*Dbi*	Diazepam-binding inhibitory peptide	1.517
*Scg5*	Secretogranin V	1.259
*Igf2*	Insulin-like growth factor 2	1.148
*Chga*	Chromogranin A and related peptides	1.128
*Chgab*	Chromogranin B and related peptides	1.118
*Vgf*	Nerve growth factor inducible protein and related peptides	1.110
*Nxph3*	Neurexophilin-3	1.012
*Scg3*	Secretogranin III	0.738
*Pthlh*	Parathyroid hormone-related proteins	0.324
*Sst*	Somatostatins	0.221

*Gene is the gene symbol; *peptide* is the known peptide(s) derived from the gene; *fold change vs. gray matter* is the degree of neuropeptide expression compared to overall gray matter in the brain*.

**Table 3:** Neuropeptides in the PVT with known roles in reproduction, as identified with the Allen Brain Atlas.

Reproduction neuropeptides		
Gene	Peptide	Fold change vs. gray matter
*Gnrh1*	Gonadotropin-releasing hormone	0.764
*Trh*	Thyrotropin-releasing hormone	0.752
*Prl2c2*	Prolactin-2c2	0.434

*Gene is the gene symbol; *peptide* is the known peptide(s) derived from the gene; *fold change vs. gray matter* is the degree of neuropeptide expression compared to overall gray matter in the brain*.

### Tachykinin 2/Neurokinin B

Tachykinin 2 (*Tac2*) is very highly expressed in the PVT, being found in numerous cells in this nucleus, albeit at low-to-moderate levels in those cells (Allen Institute, 2004). While generally consistent across the antero-posterior axis of the PVT, tachykinin 2 expression is somewhat higher in the posterior PVT, where it is denser in the medial part of this subregion (Allen Institute, 2004). Prior research using *in situ* hybridization in rats had reported that only a few cells express this gene in the PVT (Lucas et al., 1992) and noted that expression was similar between rats and mice in the thalamus as a whole (Duarte et al., 2006). It may be that the lower gene expression in individual cells was below the threshold of detection in these earlier studies. Work with immunohistochemistry has consistently identified moderate levels of the derived peptide, neurokinin B, in fibers of the PVT and in the overall thalamus of both rats and mice (Lucas et al., 1992; Marksteiner et al., 1992; Duarte et al., 2006).

Neurokinin B, the peptide encoded by *Tac2*, is a member of the larger tachykinin family, which also includes substance P and neurokinin A, which are derived from *Tac1*. Neurokinin B acts preferentially at the neurokinin receptor 3 and is perhaps best known for its role in growth and reproduction, which occur primarily *via* the hypothalamic-pituitary-gonadal axis (Zhang et al., 2020). While no prior research has examined the function of neurokinin B in the PVT, a body of work has examined the effects of neurokinin receptor 3 manipulations through injections into the lateral ventricles or periphery. In both rats and mice, stimulation of neurokinin receptor 3 induces positive hedonic motivation. Injection of a neurokinin receptor 3 agonist into the lateral ventricles of rats can, on its own, induce conditioned place preference (Ciccocioppo et al., 1998). In mice, it increases time spent and the number of entries into the open arms of an elevated plus-maze (Ribeiro and De Lima, 1998), and in rats, systemic injection of a neurokinin receptor 3 agonist increases time spent in the center of an open field (Schäble et al., 2010), which together suggest that neurokinin B is anxiolytic. Similarly, systemic injection in rats of a neurokinin receptor 3 agonist reduces immobility time in a forced swim test (Schäble et al., 2010), suggesting that it is also anti-depressive. Perhaps due to the rewarding effects of neurokinin receptor 3 stimulation, agonists of this receptor have consistently been found to reduce ethanol drinking, when injected into the lateral ventricles of selectively-bred alcohol-preferring rats (Perfumi et al., 1991; Ciccocioppo et al., 1994, 1998). In light of the higher expression of tachykinin 2 in the posterior and medial PVT, and the known ability of posterior PVT stimulation to affect anxiety-like behavior (Barson et al., 2020) and ethanol drinking (Pandey et al., 2019), as well as the involvement of the PVT in depression-like behavior (Kasahara et al., 2016; Kato et al., 2019), we hypothesize that tachykinin 2/neurokinin B in cells in the PVT could promote a positive affective state and reduce the intake of drugs of abuse.

### Galanin

Although the overall gene expression of galanin is lower than that of tachykinin 2 in the PVT, it occurs at higher levels in individual cells of this nucleus (Allen Institute, 2004). With galanin cells being most dense in the anterior PVT and becoming progressively less dense across the antero-posterior axis, galanin expression in the most anterior PVT is found more in the lateral part of this subregion but becomes more medially restricted in posterior portions of the PVT (Allen Institute, 2004). Prior research in mice using *in situ* hybridization similarly reported more abundant expression of galanin in the anterior compared to posterior PVT and in more medial parts of the PVT (Gao et al., 2020), although work with immunohistochemistry reported that galanin peptide-expressing neurons could be found at moderate levels throughout the antero-posterior axis of the PVT (Perez et al., 2001). No published research has identified galanin in the PVT of the rat.

There is substantial prior research on the behavioral role of galanin, using injections into the lateral ventricles or global gene knockdown or overexpression, but very recent research on galanin has examined it specifically in cells of the PVT itself. This work has found that galanin-containing PVT neurons in the mouse decrease their activity, as measured by calcium transients, during the transition from NREM sleep to wakefulness, and that chemogenetic activation of these cells decreases wakefulness (Gao et al., 2020). Thus, galanin in cells of the PVT appears to be important in suppressing arousal. It is possible, however, that these cells also affect valence and motivated behavior. Injection of galanin into the lateral ventricles in mice increases time spent in the open arms of an elevated zero maze (Rajarao et al., 2007), indicating that it is anxiolytic. On the other hand, galanin does not appear to play a role in depression-like behaviors, as injection of galanin into the lateral ventricles does not affect behavior in a mouse tail suspension test or rat forced swim test (Rajarao et al., 2007). Galanin does, however, have clear effects on food and alcohol intake and this could occur, in part, *via* release from the PVT. Injection of galanin into the lateral or third ventricle in rats stimulates the intake of standard laboratory chow (Koegler et al., 1999; Schusdziarra et al., 2008) and a palatable cookie mash (Crawley et al., 1990). Similarly, mice that overexpress galanin eat more of a high-fat diet than wild-type mice (Karatayev et al., 2009), while knockout or targeted deletion of galanin leads them to eat less (Adams et al., 2008; Karatayev et al., 2010). This same relationship is found with alcohol: injection of galanin into the third ventricle in rats stimulates ethanol drinking (Lewis et al., 2004), overexpression leads mice to drink more ethanol than wild-type mice (Karatayev et al., 2009), and knockout leads them to drink less (Karatayev et al., 2010). In light of the higher expression of galanin in the anterior PVT, and the known ability of anterior PVT stimulation to affect arousal (Barson et al., 2020) and promote both food intake (Cheng et al., 2018) and ethanol drinking (Barson et al., 2015, 2017), we hypothesize that galanin in cells of the PVT, in addition to suppressing arousal, could also promote motivated behavior.

### Cholecystokinin

Cholecystokinin (CCK) is present in the PVT, albeit at low levels, despite being found at high levels in many of the surrounding nuclei of the thalamus (Allen Institute, 2004). In the mouse, its gene expression is greatest in the anterior PVT, primarily in the lateral and ventral parts here, and it is similarly located in the ventral part of the posterior PVT (Allen Institute, 2004). This contrasts somewhat with published literature on the rat. Here too, gene expression of CCK, identified with *in situ* hybridization, was reported to be low but present in the PVT, in contrast to the high levels found in other areas of the thalamus (Burgunder and Young, 1988), and CCK peptide, identified with immunohistochemistry, was found to be present in cell bodies in this nucleus (Ingram et al., 1989). However, CCK in the rat, as identified with *in situ* hybridization, was found only in the posterior but not anterior PVT (Burgunder and Young, 1988). Through retrograde tract-tracing, these CCK cells were found to project to the frontal cortex and striatum (Burgunder and Young, 1988). Thus, CCK is present in the PVT, but its subregional distribution may differ across species.

Perhaps best known as a gut hormone, CCK functions in the digestive tract, in the heart, as a growth factor, as an anti-inflammatory cytokine, and as a neurotransmitter, among others (Rehfeld, 2017). It has a well-established role as an anorexigenic neuropeptide (Rehfeld, 2017), although its ability to suppress food intake appears to occur largely through actions in the hypothalamus and hindbrain, rather than through extra-hypothalamic areas of the limbic system (Blevins et al., 2000). While no published literature has reported on the role of CCK in cells of the PVT, research has examined the effects of this neuropeptide in the prefrontal cortex and nucleus accumbens, where it is believed to be released from the PVT (Burgunder and Young, 1988). Extracellular peptide levels of CCK, as measured by microdialysis, are increased in the rat prefrontal cortex in response to acute or chronic stress (e.g., restraint, injection with yohimbine, and chronic social defeat; Nevo et al., 1996; Becker et al., 2001). Levels are also increased in both the prefrontal cortex and nucleus accumbens of the rat in response to the acute or chronic administration of drugs of abuse, including morphine and cocaine (Becker et al., 1999, 2000; Beinfeld et al., 2002). Conversely, a rise in CCK levels in these areas can alter affective and motivated behavior. Injection of CCK-B into the mouse medial prefrontal cortex induces anxiety- and depression-like behavior, decreasing time spent in the open arms of an elevated plus-maze and inducing social avoidance in a social interaction test, while blockade of the CCK-B receptor in this same brain region induces a resilient phenotype in mice subjected to chronic social defeat stress (Vialou et al., 2014). In the nucleus accumbens, stimulation of CCK-B receptors leads rats to increase self-administration of the drug, amphetamine (Bush et al., 1999). Thus, CCK appears to induce an aversive state, which in turn can promote drug intake (Koob and Le Moal, 2005). As CCK in rats is found to be more highly expressed in the posterior PVT, and stimulation of this subregion affects anxiety-like behavior (Barson et al., 2020) and the intake of drugs of abuse (Pandey et al., 2019; Matzeu and Martin-Fardon, 2020), we hypothesize that CCK in cells of the PVT could promote anxiety- and depression-like behavior and promote the intake of drugs of abuse.

### Neurotensin

Neurotensin is present in cells throughout the PVT. In the mouse, its expression is most dense in the anterior PVT, decreases across the antero-posterior axis, but increases again in the most posterior region of this nucleus (Allen Institute, 2004). Neurotensin cells are most highly concentrated in the medial part of the PVT, and they are primarily dorsal, especially in the posterior PVT (Allen Institute, 2004). Prior research in rats had also identified neurotensin throughout the PVT, with both quantitative real-time PCR identifying gene expression and immunohistochemistry identifying neurotensin^+^ cell bodies in both the anterior and posterior halves of this nucleus (Arluison et al., 1994; Pandey et al., 2019; Pandey and Barson, 2020). With retrograde tract-tracing, these neurotensin^+^ cells have been found to project to the BNST (Arluison et al., 1994), although they likely project to other brain regions as well.

The majority of research on neurotensin has investigated its behavioral role in the ventral tegmental area (Torruella-Suarez and Mcelligott, 2020), but recent research from our laboratory has investigated it in the PVT, and other published literature has investigated it in the BNST and lateral ventricles. Using vertical time in a novel activity chamber to identify rats prone to higher levels of ethanol drinking, we found that gene expression and peptide levels of neurotensin specifically in the posterior but not anterior half of the PVT were reduced in these prone rats while they were still ethanol-naïve (Pandey et al., 2019). Moreover, injection of neurotensin into the posterior PVT reduced both vertical time in a novel activity chamber and high-level ethanol drinking, while a non-selective neurotensin receptor antagonist did the reverse (Pandey et al., 2019). Neurotensin here did not affect locomotor activity in a familiar activity chamber (Pandey et al., 2019). Together, these data suggest that neurotensin in cells of the posterior PVT inhibits both exploratory behavior and ethanol drinking. In the BNST, where PVT neurotensin is known to project (Arluison et al., 1994), injection of a non-selective neurotensin receptor antagonist in rats increases time spent in the open arms of an elevated plus-maze following chronic unpredictable stress, although it does not affect time immobile in a forced swim test (Normandeau et al., 2018). Thus, neurotensin release in the BNST appears to promote anxiety-like but not depression-like behavior. With injection into the lateral ventricles, neurotensin has been found to reduce food intake in rats in both a fed and fasted state (Cooke et al., 2009) and to induce reinstatement of cocaine- but not sucrose seeking in rats trained to self-administer, and then having undergone extinction training, for these substances (Lopak and Erb, 2005). Altogether, the literature suggests that neurotensin in cells of the posterior PVT alters affective behavior and the intake of drugs of abuse, but that neurotensin in the PVT could also promote relapse to drug-seeking.

### Adenylate Cyclase Activating Polypeptide 1/Pituitary Adenylate Cyclase-Activating Polypeptide

Adenylate cyclase activating polypeptide 1 (*Adcyap1*) is also expressed in cells of the PVT, being located across its antero-posterior axis (Allen Institute, 2004). Cells expressing this gene in the mouse are slightly more dense in the anterior compared to posterior PVT, and they are in the more lateral part throughout this nucleus (Allen Institute, 2004). Prior research in mice, using immunohistochemistry, had reported relatively uniform expression of the derived peptide, pituitary adenylate cyclase-activating polypeptide (PACAP), across the antero-posterior axis of the PVT (Gupta et al., 2018). While published literature in rats on *Adcyap1*, using *in situ* hybridization and quantitative real-time PCR, had similarly identified this neuropeptide throughout the PVT (Murase et al., 1995; Skoglösa et al., 1999; Gupta et al., 2018), research using immunohistochemistry identified more PACAP^+^ cells in the posterior compared to anterior half of this nucleus, identified PACAP-27 rather than PACAP-38 as being the predominant isoform here, and found that PACAP-27^+^ cells are more concentrated in the medial rather than lateral PVT (Gupta et al., 2018). Tract tracing has not yet been used to determine the projections of these PACAP^+^ PVT cells; however, given the dense anatomical projections of the PVT to the nucleus accumbens shell and BNST (Dong et al., 2017), there is a high likelihood that PVT PACAP^+^ cells also project to these brain regions.

A pleiotropic peptide, PACAP is well-known for its role in regulating the stress response and it is increasingly recognized for its role in motivated behavior (Gargiulo et al., 2020a). Recent research from our laboratory has found that PACAP in the PVT is stimulated by ethanol drinking. Specifically, ethanol drinking in rats leads to a significant increase in levels of PACAP-27 in individual cells across the PVT (Gupta et al., 2018). In the nucleus accumbens shell, where this PACAP is likely released, injection of PACAP-27 in turn specifically reduces ethanol drinking, while a PACAP receptor antagonist instead increases it (Gargiulo et al., 2020b). These same injections do not affect sucrose drinking or anxiety-like behavior, as measured in a light-dark box and open field (Gargiulo et al., 2020b). These data together suggest that a major role of PACAP in the PVT is the regulation of ethanol drinking *via* negative feedback. Through its projections to the BNST, in contrast, PACAP may instead be more involved in promoting a negative affective state and a relapse to drug-seeking. Notably, in rats, injection of a PACAP receptor agonist in the BNST reduces time spent in the open arms of an elevated plus-maze and in the center of an open field (Roman et al., 2014), suggesting that PACAP release here induces anxiety-like behavior. Interestingly, in rats trained to self-administer cocaine and then having undergone extinction training, injection of PACAP into the BNST increases seeking for cocaine, while injection of a PACAP receptor antagonist blocks footshock-induced reinstatement of cocaine-seeking (Miles et al., 2018). In ethanol-dependent rats, injection of a PACAP receptor antagonist into the BNST blocks excessive ethanol intake and withdrawal-induced anxiety-like behavior in a light-dark box (Ferragud et al., 2020). Thus, PACAP in cells of the PVT may have a different relationship with affective and motivated behavior depending on its site of release. While PACAP activity in the nucleus accumbens shell reduces drug intake but has no effect on anxiety-like behavior, PACAP in the BNST instead promotes drug seeking and intake as well as anxiety-like behavior. It remains to be determined if these varied effects can all originate from PACAP^+^ cells of the PVT.

### Prodynorphin/Dynorphins

Prodynorphin is expressed relatively uniformly throughout the PVT, at low-to-moderate levels in individual cells, with expression decreasing across the antero-posterior axis (Allen Institute, 2004). Prior research using *in situ* hybridization in mice had identified a few prodynorphin cells in the PVT (Lin et al., 2006), confirming the presence of prodynorphin in this nucleus. Older literature using *in situ* hybridization in rats had failed to identify prodynorphin gene expression in most of the thalamus (Mansour et al., 1994), so it remains to be demonstrated that rats also express this neuropeptide in the PVT.

Dynorphins, the peptides derived from prodynorphin, are members of the larger opioid family, which also includes enkephalins and endorphins. Dynorphins act preferentially at the kappa opioid receptor and are widely recognized as stress-related neuropeptides. Activation of the kappa opioid receptor generally produces negative affective states and aversion, which can contribute to the negative reinforcement that promotes drug addiction (Tejeda and Bonci, 2019). Indeed, this is largely what occurs when the kappa opioid receptor is stimulated in the PVT itself or in two of its major projection regions, the BNST and nucleus accumbens shell (Dong et al., 2017). Very limited research has investigated the effects of kappa-opioid receptor stimulation in the PVT. While the injection of a kappa-opioid receptor agonist in the rat PVT was found to prevent context-induced reinstatement of ethanol seeking (Marchant et al., 2010), injection of dynorphin-A into the rat posterior PVT did not affect reinstatement of seeking for cocaine or sweetened condensed milk (Matzeu et al., 2018). Thus, the effects of dynorphins in the PVT on motivated behavior may be substance- or subregion-specific. Effects of kappa receptor manipulation in the BNST, in contrast, have been more consistent. Here, injection of a kappa-opioid receptor agonist in rats stimulates reinstatement of ethanol seeking following extinction (Le et al., 2018), while blockade of these receptors attenuates the escalated ethanol self-administration that occurs during acute withdrawal in ethanol-dependent rats (Erikson et al., 2018). Similarly, both ethanol and sucrose drinking is reduced in mice by injection of a kappa-opioid receptor antagonist in the BNST (Haun et al., 2020). Thus, in the BNST, dynorphins appear to function to promote ethanol intake and reinstatement of ethanol seeking. The effects of kappa receptor manipulation in the nucleus accumbens, however, vary according to the subregion. As with effects on ethanol drinking after manipulation in the BNST, blockade of the kappa-opioid receptor in the rat nucleus accumbens shell reduces drug intake, inhibiting the increased ethanol drinking that occurs after forced abstinence (Uhari-Väänänen et al., 2019), the escalation of methamphetamine intake that occurs with extended drug access (Whitfield et al., 2015), and the escalation of heroin intake that occurs with extended drug access (Schlosburg et al., 2013). On the other hand, stimulation of the kappa-opioid receptor in the rat accumbens shell has largely opposite effects on affective behavior, depending on the subregional location of the agonist injection. In the caudal shell, a kappa-opioid receptor agonist inhibits positive orofacial reactions to an oral sucrose infusion, reduces time spent in the light chamber of a light-dark box, and reduces locomotor activity in an open field (Castro and Berridge, 2014; Pirino et al., 2020). In contrast, in the rostral shell, this same injection instead promotes positive orofacial reactions to sucrose, increases center entries into an open field, and increases locomotor activity in an open field (Castro and Berridge, 2014; Pirino et al., 2020). Thus, in the caudal nucleus accumbens shell, dynorphins induce a negative affective state and promote anxiety-like behavior, but in the rostral shell, they instead promote reward and are anxiolytic. Altogether, the literature suggests that dynorphins in the PVT, where they are expressed more densely in the anterior compared to the posterior subregion, can produce a negative affective state and promote the intake and seeking of drugs of abuse, but their effects are highly dependent on the substance being tested and the precise location in which dynorphins are released.

### Cocaine- and Amphetamine-Regulated Transcript

Cocaine- and amphetamine-regulated transcript (CART) is expressed at very high levels in cells in the most anterior aspect of the PVT, in its dorsomedial part, but it is found only at very low levels in the rest of the PVT (Allen Institute, 2004). Prior research using *in situ* hybridization in rats had reported that the CART gene was not expressed in the PVT (Douglass et al., 1995; Hurd and Fagergren, 2000). While research using immunohistochemistry in rats had identified a high density of CART-containing fibers in the PVT, primarily in the posterior half of this nucleus (Koylu et al., 1998; Kirouac et al., 2006), these fibers were afferent inputs, originating primarily from nuclei within the hypothalamus (Kirouac et al., 2006). As this prior research had been conducted in rats, the cluster of CART neurons in the anterior PVT may be found only in mice.

Major effects of CART include suppression of drug and food intake and seeking, but this neuropeptide is also involved in affective behavior, learning and memory, and osmoregulation and blood pressure (Ong and McNally, 2020). While a few studies have investigated the effects of CART in the PVT itself, this work was conducted in rats, leaving it unclear if local CART expression could contribute to the observed effects. Specifically, injection of CART or CART peptide fragments into the PVT reduces drug-primed reinstatement of cocaine-seeking (James et al., 2010) and reduces the threshold for intracranial self-stimulation, *via* an electrode in the lateral hypothalamus–medial forebrain bundle (Choudhary et al., 2018), while leaving unaffected locomotor activity in an open field (Choudhary et al., 2018). This suggests that the release of CART in the PVT enhances reward and reduces drug-seeking, without affecting arousal. Somewhat similar effects have been found with injections into the nucleus accumbens. Injection of CART into the rat nucleus accumbens reduces the number of infusions, number of active lever presses, and breakpoint for cocaine but not food (Jaworski et al., 2008), and injection into the rat accumbens shell attenuates context-induced reinstatement of ethanol seeking (Millan and Mcnally, 2012). With injection into the lateral ventricles, CART and CART peptide fragments consistently reduce food intake in both rats and mice (Lambert et al., 1998; Edwards et al., 2000; Asakawa et al., 2001; Stanley et al., 2001) and, in mice, CART also increases time spent in the closed arms of an elevated plus-maze (Asakawa et al., 2001), indicating that it enhances anxiety-like behavior. Thus, it seems likely that CART in cells of the PVT, which are largely restricted to the most anterior and medial portion of the mouse PVT, acts to reduce drug-seeking and intake and may do the same for food, but its effects on affective state remain to be delineated.

### Corticotropin-Releasing Hormone

Corticotropin-releasing hormone (CRH), also called corticotropin-releasing factor, is found across the antero-posterior axis of the PVT (Allen Institute, 2004). Cells expressing this gene are slightly more dense in the posterior compared to anterior PVT, and expression in both subregions is largely medial and also denser than in the middle antero-posterior subregion of the PVT (Allen Institute, 2004). Prior research, in CRH-IRES-Cre mice, had identified considerable numbers of CRH^+^ cells in the PVT (Peng et al., 2017), and also reported that they spanned the antero-posterior axis of the PVT, with somewhat higher density in the posterior portion of the PVT (Itoga et al., 2019). Using retrograde and anterograde viral tracing, these CRH^+^ cells were found to send a major input to the nucleus accumbens (Itoga et al., 2019), although they may project to other regions as well. Despite the generation of a CRH-Cre rat (Pomrenze et al., 2015), it remains to be demonstrated that rats also express CRH in the PVT.

Originally characterized as the central activator of the stress response, CRH is perhaps best known for its release from the paraventricular nucleus of the hypothalamus in response to stress exposure, and its subsequent triggering of the fight-or-flight response (Logrip et al., 2011). Notably, CRH^+^ cells have also been identified in extra-hypothalamic brain areas, in regions of the extended amygdala, which include the nucleus accumbens shell, BNST, and central nucleus of the amygdala (Logrip et al., 2011). In these brain regions, which receive extensive afferent input from the PVT (Dong et al., 2017), CRH is hypothesized to play a central role in the development of drug dependance, such that withdrawal from drug access increases the release of CRH, which subsequently triggers anxiety and negative affective states that prime an individual for drug relapse (Logrip et al., 2011). While extremely limited research has addressed the role of CRH in the PVT, a role for PVT CRH in negative affect and drug use would be consistent with the known functions of both CRH and the PVT in motivated behavior. Indeed, work suggests that CRH in cells of the PVT could induce anxiety-like behavior, as an injection into the rat lateral ventricles of a CRH receptor antagonist can reverse the reduced open arm time and the number of entries in an elevated plus-maze that is triggered by injection into the posterior PVT of the neuropeptide, orexin (Li et al., 2010). With CRH^+^ cells known to project from the PVT to the nucleus accumbens, similar results have been found with injections in this latter brain region. Specifically, injection of CRH into the rat nucleus accumbens shell reduces time spent in the open arms of an elevated plus-maze and in the center area of an open field (Chen et al., 2012). These same injections also promote depressive-like behavior, reducing preference for sucrose in a sucrose preference test, and increasing time immobile in a forced swim test (Chen et al., 2012). Paradoxically, CRH activity in the nucleus accumbens can also promote reward, as drug-primed reinstatement of morphine conditioned place preference in rats is blocked by prior injection into the nucleus accumbens of a CRH receptor antagonist (Wang et al., 2006). Finally, consistent with the idea that CRH in the extended amygdala can promote relapse to drug-seeking, injection of CRH into the nucleus accumbens stimulates ethanol drinking in ethanol-preferring P rats, and injection of a CRH receptor antagonist blocks the stress-induced increase in ethanol drinking (Knapp et al., 2011). Altogether, the literature suggests that CRH, which is expressed more densely in cells of the medial posterior rather than anterior PVT, can promote anxiety and produce a negative affective state and, subsequently, *via* projections to the nucleus accumbens, promote the intake and seeking of drugs of abuse.

### Proenkephalin/Enkephalins

Proenkephalin is located in cells across the antero-posterior axis of the PVT, primarily in the medial PVT, with its expression most dense in the anterior aspect of this nucleus but also somewhat dense in the posterior aspect (Allen Institute, 2004). Prior research using *in situ* hybridization in rats had identified only a few proenkephalin cells in the PVT (Hermanson et al., 1995), but research using immunohistochemistry had reported moderate numbers of soma containing enkephalin peptides (Wamsley et al., 1980; Merchenthaler et al., 1986; Abe et al., 1987; Arluison et al., 1994). Moreover, consistent with the *in situ* hybridization results in mice (Allen Institute, 2004), these immunohistochemistry results in rats showed that enkephalin^+^ cells are denser in the anterior compared to posterior PVT (Abe et al., 1987). With retrograde tract-tracing, these enkephalin^+^ cells have been found to project to the BNST (Arluison et al., 1994), although they likely project to other brain regions as well.

Enkephalins, the peptides derived from proenkephalin, are members of the larger opioid family, much like dynorphins. Enkephalins act through both mu and delta-opioid receptors and, in contrast to dynorphins, generally produce positive affective states, which can contribute to positive reinforcement (Le Merrer et al., 2009; Henry et al., 2017). Acting through various brain regions, enkephalins can affect a wide range of physiological processes and behaviors, including but not limited to feeding, thirst, reward, drug use, anxiety-like behavior, and analgesia (Le Merrer et al., 2009; Henry et al., 2017). They also appear to reduce fear expression and promote locomotor activity. Very recent research has demonstrated with an injection of a mu-opioid receptor agonist in the mouse anterior PVT that mu-opioid stimulation reduces freezing and promotes locomotor activity during recall of Pavlovian fear conditioning extinction, while injection of a mu-opioid receptor antagonist instead maintains freezing during fear extinction sessions (Bengoetxea et al., 2020). This apparent ability of enkephalins in the PVT to promote locomotor activity is consistent with their effects in other regions of the brain, as injection of mu and delta-opioid receptor agonists into the lateral ventricles of the mouse promotes horizontal locomotor activity (Mickley et al., 1990). The apparent ability of enkephalins in the PVT to reduce the expression of conditioned fear behavior likely occurs, in part, through their projections to the BNST. Notably, hypothalamically-elicited hissing behavior in the cat is inhibited by injection of a mu and delta-opioid receptor agonist into the BNST (Brutus et al., 1988). Thus, with enkephalins expressed most densely in cells of the medial anterior PVT, they appear to play a role in promoting arousal and inhibiting fear behavior, and they likely affect behaviors well beyond what has so far been explored.

### Comparison: Glutamate and GABA

For comparison with the neuropeptides, we herein describe the distribution of glutamate and γ-aminobutyric acid (GABA) in the PVT. A biochemical marker of glutamatergic neurons, *Slc17a6*, the gene that encodes vesicular glutamate transporter 2 (vGLUT2), is expressed at very high levels in cells across the antero-posterior axis of the PVT (Allen Institute, 2004). In contrast, the gene that encodes vesicular glutamate transporter 1 (vGLUT1), *Slc17a7*, is expressed only at very low levels in this nucleus (Allen Institute, 2004). Prior research using *in situ* hybridization in rats had identified significant gene expression vGLUT2 but none of vGLUT1 in the PVT (Barroso-Chinea et al., 2007), although research using radioimmunocytochemistry in rats has detected vGLUT1 protein in this nucleus (Boikess et al., 2010). Similarly, in vGLUT2-GFP transgenic mice that express a green fluorescent protein in glutamatergic cells, nearly two-thirds of all cells have been found to contain vGLUT2 across the antero-posterior axis of the PVT (Huang et al., 2006; Gupta et al., 2018) and, with *in situ* hybridization in rats, nearly all prefrontal-cortex-projecting cells in the PVT have been found to contain vGLUT2 (Hur and Zaborszky, 2005). Thus, glutamate is densely expressed across the PVT, and vGLUT2 is the predominant glutamate transporter in this nucleus.

While a biochemical marker of GABAergic neurons, *Gad1*, is not detectable in the PVT, another marker for GABAergic neurons, *Gad2*, is present in the dorsolateral part of the most anterior portion of the PVT (Allen Institute, 2004). This is at odds with published literature on GABA expression which, using *in situ* hybridization in rats, reported that *Gad* was not present in the thalamus, beyond the reticular nucleus and lateral geniculate body (Feldblum et al., 1993) and, using immunohistochemistry in mice and rats, reported that there were no GABA^+^ cell bodies in the midline thalamus (Ottersen and Storm-Mathisen, 1984). Interestingly, a finding of GABA in cells of the anterior PVT is consistent with work using whole-cell recordings in rats, which reported that stimulation of the anterior PVT resulted in both excitatory and inhibitory postsynaptic potentials in the suprachiasmatic nucleus, and concluded that both glutamate and GABA are released from the PVT (Alamilla and Aguilar-Roblero, 2010). Thus, in a circumscribed region, GABA does appear to be expressed in the PVT and the cells expressing this neurotransmitter may be projection neurons.

## Conclusions and Future Directions

Several themes emerge from the investigation of the pattern of neuropeptide expression across the PVT. First, while the majority of neuropeptides are expressed across the antero-posterior axis of the PVT, they generally exist in a gradient, in which expression is most dense in either the anterior or posterior PVT and gradually reduces across the rest of the nucleus. Other neuropeptides, however, including neurotensin, CRH, and proenkephalin, display somewhat more equal expression in the anterior and posterior PVT but have reduced expression in the middle PVT. Second, neuropeptides involved in arousal, including galanin and proenkephalin, appear to be more highly expressed in the anterior PVT; those involved in depression-like behavior, including tachykinin 2, cholecystokinin, and CRH, are more highly expressed in the posterior PVT; those involved in reward, including tachykinin 2 and cocaine- and amphetamine-regulated transcript, are more highly expressed in the medial PVT; but those involved in the intake of food and drugs of abuse are distributed throughout the PVT. Third, the pattern and content of neuropeptide expression in mice and rats may not be identical. Clear differences in the pattern of expression exist with cholecystokinin and adenylate cyclase-activating polypeptide 1 and several neuropeptides, including tachykinin 2, galanin, prodynorphin, cocaine- and amphetamine-regulated transcript, and CRH, have yet to be demonstrated in the rat. It remains to be determined if newer technologies can identify these neuropeptides in the rat PVT or if the rat PVT simply contains fewer neuropeptides.

Many questions remain regarding the expression and function of neuropeptides in the PVT. While the Allen Brain Atlas has allowed us to identify and thoroughly describe several neuropeptides in cells of the PVT in the male mouse, the same needs to be established for the female mouse and the male and female rat, and the projections of these specific cell populations remain to be determined. Indeed, it has recently been argued that neurons in the PVT are dissociable based on gene expression and circuit connectivity, as well as anatomical location and activity dynamics (McGinty and Otis, 2020), and this may be especially relevant to neuropeptides involved in the intake of food and drugs of abuse, which are less dissociable by PVT subregion. In a similar vein, the afferent input to these neuropeptide-expressing cells in the PVT should be determined, as they may receive input that is anatomically and functionally selective. It has also not been established to what extent the neuropeptides are expressed in neurons compared to glia in the PVT. It is also unclear if and to what extent the neuropeptides are co-expressed with each other in the PVT. It has been established in the hypothalamus that the neuropeptides orexin and dynorphin are packaged in the same synaptic vesicles and are thus co-released, despite having opposite effects on behavior (Muschamp et al., 2014). We postulate that the same co-expression and co-release could also occur with neuropeptides in the PVT. For example, with tachykinin 2 reducing anxiety-like behavior and CRH promoting it, it is notable that both neuropeptides are most densely expressed in the posterior and medial PVT. It is possible that these neuropeptides are co-expressed and regulate the behavioral effects of each other, much like orexin and dynorphin. Importantly, the behavioral functions of the neuropeptides in the PVT have only recently begun to be described. Future studies should apply genetic and pharmacological techniques for neuropeptide-specific monitoring and manipulation in the PVT, to delineate the specific function of the neuropeptides in the PVT itself. Studies should also involve projection-specific manipulations, using optogenetic and chemogenetic approaches, to precisely manipulate the specific neuropeptide projections to other brain regions.

While significantly more work is required to uncover the expression patterns and specific roles of the individual neuropeptides, including those identified but not discussed in the present review article (see Tables s 1–3), the evidence thus far supports a diverse yet highly organized system of neuropeptides in the PVT. Determined in part by their location within the PVT and their network of projections, the function of the neuropeptides in this system likely involves intricate coordination to influence both affective and motivated behavior.

## Author Contributions

GC, KO, and JB wrote and edited the manuscript. All authors contributed to the article and approved the submitted version.

## Conflict of Interest

The authors declare that the research was conducted in the absence of any commercial or financial relationships that could be construed as a potential conflict of interest.
